# Smartphone Use during the COVID-19 Pandemic: Social Versus Physical Distancing

**DOI:** 10.3390/ijerph18031034

**Published:** 2021-01-25

**Authors:** Meredith E. David, James A. Roberts

**Affiliations:** Department of Marketing, Hankamer School of Business, Baylor University, Waco, TX 76798, USA; jim_roberts@baylor.edu

**Keywords:** social distancing, physical distancing, COVID-19, social connection, smartphone use, pandemics, well-being

## Abstract

The COVID-19 pandemic continues to wreak havoc across the globe. According to the Centers for Disease Control and Prevention, limiting face-to-face interaction is the best strategy for reducing the spread of COVID-19. We investigate the impact of social distancing on social connection and well-being, while also considering the moderating influence of smartphone use. In a survey of 400 students, the study presented herein finds that smartphone use attenuates the negative impact of social distancing on social connection and well-being. Contrary to popular sentiments regarding the influence of smartphone use on well-being, increased smartphone use during the pandemic may foster social connection and well-being. Overall, the research presented provides evidence that the perceived loss of social connection with others is not a de facto outcome of social distancing. The study’s findings have important implications for public policymakers, government officials, and others, including consumer researchers. These implications include stressing the important role technology can play in staying socially connected during the current pandemic and the importance of reframing “social distancing” as “physical distancing with social connectedness”.

## 1. Introduction

The current COVID-19 pandemic continues to wreak havoc across the globe. As of 29 December 2020, there have been 19,055,869 COVID-19 cases diagnosed in the US and 82,086,503 worldwide. To date, 332,246 have died in the US and 1790,597 worldwide as a result of the Coronavirus pandemic [1,2]. According to the US Centers for Disease Control and Prevention (CDC), limiting face-to-face contact with others is the best strategy for reducing the spread of the Coronavirus disease [3]. Social distancing, providing a safe buffer between ourselves and others outside of our homes, can take on many forms including: (1) staying at least six feet (about 1.8 m) apart from other people, (2) working from home, (3) limiting the size of social gatherings, (4) not using public transportation, (5) avoiding crowded places and large gatherings, (6) sheltering in place, and (7) self-quarantining if one suspects they may have the disease or have been exposed to someone who does. Schools have transitioned to online learning, businesses have closed, many cities and states at one point have issued shelter-in-place orders, and airline travel has declined significantly [4].

Strict social distancing may be the best way to avoid contracting or spreading the COVID-19 disease, but it only addresses our physical well-being. Our overall well-being must consider our mental health as well. The negative impact of the COVID-19 pandemic on our mental health is beginning to emerge. US adults are reporting significant increases in symptoms of psychological distress. One survey found that 13.6% of adults (compared to 3.9% in 2018) reported serious psychological problems. It appears that adults between the ages of 18 to 29 are suffering the most from the pandemic with 25 percent reporting significant psychological distress [5]. Many of those surveyed reported feeling socially isolated, lonely, stressed, anxious, and depressed because of the pandemic [6]. Social connectedness is best understood as the extent to which an individual feels an emotional bond with others [7]. Feeling connected to others is a reward for people who foster and maintain social connections and often leads to a heightened sense of well-being [8].

A lack of a sense of social connection to others undermines our mental well-being. Jamil Zaki [9] and other health-care professionals [6,10] have argued that we must reframe how we approach social distancing. Zaki argues that we are practicing “physical distancing” rather than “social distancing” to highlight the need for people to remain socially connected even while keeping their physical distance from others.

### 1.1. Study Objectives

The primary objective of the present study is to investigate the impact of social distancing on well-being. Given the importance of social connection to human well-being [11,12,13], social distancing would likely undermine an individual’s sense of being socially connected to others and ultimately reduce one’s psychological well-being. This line of thinking, however, may not hold during the current pandemic for two possible reasons. First, technology may allow individuals to remain in touch with those from who they are currently physically distancing. Staying at home may provide individuals with the time to connect or reconnect with meaningful others who were not part of a person’s everyday routine prior to the pandemic. Stories abound about family dinners, dates, gatherings on Zoom, calling friends and family to talk rather than sending an impersonal text, and getting the chance to interact with one’s neighbors since the pandemic began [14]. The pandemic may have reminded us of the importance of relationships [15]. The smartphone, often derided as something that has alienated us from others [16,17], might very well be the tool that allows us to connect or reconnect with family, friends, coworkers, and others [9]. The present study investigates the role smartphone use plays regarding the relationship between social distancing and social connection. 

### 1.2. Social Distancing, Social Connection, and Well-Being

Maintaining social distance from others runs counter to human well-being [6]. Humans have a fundamental need to connect with others. In fact, the lack of social connections is tantamount to such health risks as smoking cigarettes or not exercising [18]. Van Orden et al. [19] argue that social changes at the macro level (e.g., the current pandemic) can have a negative impact on individual well-being. The authors use the hypothesis, *Is society “pulling together” or “pulling apart”?* to explain how societal-level changes can impact individual well-being. For example, the terrorist attacks on 9/11 pulled together US citizens as they came together to fight a common foe. These types of events may bring people closer together. On the other hand, tragedies or events that create social distancing (such as the current pandemic) may be associated with “pulling-apart” communities and individuals. Such “pulling apart” has been found to increase mental health problems [19].

Using a survey of college students, Bian and Leung [20] found that loneliness was the most powerful predictor of both bonding and bridging capital. Loneliness has been consistently found to be related to decrements in both physical and mental well-being [21]. When we feel a lack of connection with others, we are more likely to exhibit signs of depression, stress, and anxiety [19].

Reframing social distancing, however, may change the direction of its relationship with social connection and well-being. As called for by Zaki [9] and others [22], reframing “social distancing” to “physical distancing” could potentially change its influence on an individual’s sense of social connection and well-being. Social distancing suggests breaking off our connection with others while “physical distancing” connotes that we must keep our physical distance from others, but we need not become socially distant. Ironically, the smartphone and other technologies that have been criticized for separating us from our fellow humans [16,20,23] may now be the best tools available to maintain or grow our social connections. 

### 1.3. The Moderating Influence of Smartphone Use on the Social Distancing-Social Connection Relationship

As argued above, social distancing can undermine an individual’s sense of social connection and have a negative effect on one’s psychological well-being. During the current pandemic, however, technology may enable individuals to maintain their social connections despite physical distancing. Smartphones allow us to call, text, direct message, shop, surf the internet, and scroll (or post, comment, and like) our social media feeds. A small body of research has found that social media use can help individuals build bridging, bonding, and maintained social capital [24,25,26]. In a national survey of Chinese adults, Chan [24] found that mobile phone use was positively associated with both bridging and bonding capital as well as psychological and emotional well-being. An experiment conducted by Deters and Mehl [25] asked subjects in the treatment group to make more posts than usual to Facebook. Results of the experiment found that this increased posting led to lower levels of reported loneliness. These same subjects reported that they felt more connected to their friends because of their increased posting. In a large (n = 2708) sample of South Korean adults, Cho [27] found that the use of communication apps on smartphones was associated with lower levels of reported loneliness and enhanced feelings of social capital. Such social capital can reduce loneliness and enhance psychological well-being [28]. Research by Primack et al. [21] and Verduyn et al. [29] found that social media use may foster social connection by providing avenues for social support. Wei and Lo [30] p. 53 argue that smartphone use can enhance social connection providing “instant membership in a community”.

Given the current circumstances surrounding the COVID-19 crisis and call for social distancing, the smartphone and other technologies may moderate the otherwise negative influence of social distancing on our sense of social connection and ultimately, our psychological well-being. Next, we present a study designed to test the proposed moderating effect of smartphone use on the social distancing-social connection relationship.

## 2. Materials and Methods

We designed and conducted a survey to assess the relationship between social distancing and social connection, as well as well-being. The study also examines the role of smartphone use, in the relationship between social distancing and feelings of social connection, to explore whether the predicted negative association between distancing and feelings of connection is attenuated by smartphone use. 

### Participants and Procedures

The sample consisted of 400 undergraduate students from a large U.S. university (52% female, M_age_ = 20). Students completed the study in exchange for course credit. The majority of participants were sophomores (i.e., 62% were in their second year of university studies), followed by juniors (28%) and then seniors (9%) and freshman (1%). The ethnicities of the sample were as follows: 75% white/Caucasian, 11% Hispanic/Latino, 8% Asian/Pacific Islander, 4% black/African American, and 2% other. The survey was completed online through Qualtrics in exchange for course credit. The independent variable, social distancing (α = 0.92), was measured using two items developed for the current study in which respondents indicated their agreement on a seven-point Likert scale to the following statements concerning their response to the Coronavirus outbreak: “I have strictly practiced social distancing” and “I have done my very best to quarantine myself from other people”. 

The mediator, social connection (α = 0.89), was assessed using the Lee and Robbins [31] measure; example items include “I feel distant from people” and “I have no sense of togetherness with my peers”. A seven-point Likert scale was used to assess the items. The moderator, phone usage, was assessed using an objective self-report directly from participants’ iPhones. Specifically, participants who have an iPhone were asked to follow several steps on their iPhone which involved going to Settings and Battery information to find and report the exact amount of “Screen On” time. Several participants reported not having an iPhone, resulting in a final sample size of 378 for the study analyses.

The dependent variable, well-being (α = 0.87), was assessed using two items (e.g., “Overall, I am satisfied with my life”) in which participants indicated their agreement on a seven-point scale. The study also included a measure of negative well-being (α = 0.94) which was assessed using the four-item measure (PHQ-4) of depression and stress developed by Kroenke and colleagues [32] and used in related work by David and Roberts [33]. Descriptive statistics and correlations between the study variables are provided in Table 1.

## 3. Results

The Preacher and Hayes [34] PROCESS macro for SPSS was used to run our empirical analyses. This method uses an ordinary-least-squares (OLS) path analysis to estimate model coefficients and to assess the indirect and/or direct effects of an independent variable [35]. The PROCESS models use a bootstrapping procedure (*n* = 5000), which does not rely on any assumptions about the normality of the sampling distribution, to calculate the bias-corrected 95% confidence intervals associated with the statistical significance of the indirect effects [34,35,36]. 

The Preacher and Hayes [34] PROCESS bootstrapping Model 7 was used to test whether social distancing is negatively associated with feelings of connection, which leads to lower well-being, and whether one’s use of his/her smartphone may attenuate the negative impact of social distancing. The model first tests the effects of social distancing, phone use, and the interaction of these variables on perceived social connection (*F*_3, 374)_ = 9.47, *p* < 0.10, *R*^2^ = 0.07). (Of note, social connection was measured such that higher scores corresponded to greater feelings of being disconnected). The results show that social distancing (*b* = 0.33, *p* < 0.05) and phone use (*b* = 0.27, *p* < 0.05) are associated with social connection; in addition, the interaction between social distancing and phone usage is significant (*b* = −0.03, *p* < 0.05). Specifically, the impact of social distancing on social connection is significant when phone use is low (*b* = 0.23, *p* < 0.05), but becomes non-significant when usage is high (*p* > 0.05). 

Next, the model tests the impact of social distancing and social connection on subjective well-being (See Table 2 for full results). The main effect of social distancing is non-significant, but the effect of social connection is significant (*b* = −0.29, *p* < 0.05). Importantly, the results show support for moderated mediation such that the indirect effect of social distancing on well-being (through social connection) is significant, but only among individuals who had lower phone use (*b* = −0.06, *p* < 0.05). These results indicate that social distancing during the current pandemic is harmful to one’s subjective well-being unless individuals actively use their phones, likely to engage with and interact with others virtually. Similar results were found for the depression and stress measures of well-being.

Overall, the results show that phone use mitigates the negative impact of social distancing on feelings of connection, and ultimately well-being. In addition, the results provide evidence that could suggest that the negative effects of social distancing may be explained by the way in which individuals experience social distancing, albeit either focusing on being socially distanced such as not being able to see friends or focusing on being physically distanced but yet being able to socially connect or reconnect with meaningful others.

## 4. Discussion

As humans, we are social animals and have a primordial need to socially connect with others. Social distancing during the current COVID-19 pandemic on its surface appears to interfere with our basic need to connect with others. The current study, however, provides evidence that the perceived loss of social connection with others is not a de facto outcome of social distancing. 

Our study found that social distancing is negatively associated with social connection. The more we socially distance ourselves from others the more socially disconnected we feel. Given the importance of social connection, this lack of social connection was found to be associated with higher reported levels of stress and depression and lower subjective well-being.

Interestingly, we found that smartphone use moderates the relationship between social distancing and social connection. Higher use of one’s smartphone improves an individual’s perceived social connection which is associated with better psychological well-being. This finding is particularly insightful given the often-maligned impact of smartphone use on relationships and social connections prior to the pandemic [16]. We believe that the use of smartphones to connect with others is still not as effective as face-to-face interaction [8] but appears to be a suitable alternative during a public health crisis like the current COVID-19 pandemic. According to the societal “pulling together” or “pulling apart” hypothesis, societal events like the current pandemic tend to separate individuals from one another and increase mental health problems during and after the crisis [19]. People around the world have found a myriad of ways to leverage technology, including smartphones, to maintain some semblance of connection to others.

The current findings have several important implications for public policymakers, government officials, and others, including consumer researchers. First, the importance of technology in keeping socially connected must be stressed. Prior to the pandemic, smartphones were a separating force between people and others in their social network. Since the pandemic, this may no longer be the case. People need to be encouraged to use whatever technology is available to them to stay socially connected. This may be particularly difficult for the elderly and the economically disadvantaged segments of society. Computer-mediated communications are no substitute for face-to-face interactions but appear to be an adequate stopgap measure during the current crisis.

A second important implication of the current research is that something as simple as a positive reframing of social distancing can have a salubrious impact on both a sense of social connection and subjective well-being, as well as stress and depression. People must learn that even though they are being asked/require to socially distance themselves from others this does not mean they cannot stay socially connected with others. Although it may be difficult to get people to reframe in a positive manner the idea of staying physically apart from others, the undetermined length of this “new normal” may justify efforts to shift how people think about the difficult task of separating ourselves from others. Psychology professor, Jamil Zaki, suggests we should encourage people to practice “distant socializing”—remaining physically distant but maintaining social connection through technology [9]. Again, the technology that has been blamed for weakening our connections to others may, at least for the time being, be the best means of preserving our imperiled social connections.

### Limitations and Future Research Directions

Although this study is the first to examine the impact of social distancing on social connection and well-being and the moderating role of smartphone use, certain limitations do exist. Larger random samples would help in generalizing the results to the larger population or specific segments that might be particularly vulnerable to the effects of social distancing including adolescents and the elderly.

Future research into the role of technology during tragedies that necessitate social distancing like the current COVID-19 pandemic would be enlightening. With the likelihood of the return to a “new normal,” it is critical that we improve our understanding of technology’s role in decreasing the perceived psychological distance between people being asked or required to practice social distancing. Media usage during the current pandemic has risen sharply. Television viewing, internet traffic, and social media usage have all shown sizeable gains [37]. Previous research has shown that some social media platforms are more conducive to fostering connections than others [38]. Additionally, it will be helpful to investigate which individual activities on one’s smartphone are the best at enhancing social connection. Research in this area is essential because many people are spending a lot of time online and are getting their “COVID news” from online sources as well.

Lastly, more experimental and longitudinal research is needed. Although the immediate danger to our physical health of not social distancing is clear, little is known about both the short and long-term psychological effects of such pandemics. These short and long-term mental health consequences are of significant importance to individual and societal-level well-being, [39]. Wiederhold [37] p. 275 states that “…mental health issues in the surviving population can have far greater and longer lasting impacts.”

## 5. Conclusions

Utilizing a large-scale survey of US college students, the present study finds that social distancing leads to lower levels of reported social connection and well-being. This relationship, however, is moderated by smartphone use. Prior to the current pandemic, the smartphone was seen by many as hampering social connection and undermining well-being. Current results suggest that smartphones can foster social connection and ultimately, overall well-being. Important implications of the study include stressing the importance of technology like smartphones in bridging the perceived social distance between people during the COVID-19 pandemic (or other situations that require physical distancing) and reframing “social distancing” to stress that, although we must physically distance ourselves from others, this does not mean that we cannot stay socially connected with the help of modern technology.

## Figures and Tables

**Table 1:** Descriptive Statistics and Correlations between Social Distancing, Iphone.Use, Social Connection, and Well-being

Variable	*n*	M	SD	Range	1	2	3
Distancing	400	5.10	1.28	1–7			
Phone Use	378	5.37	2.94	1–17	0.077		
Connection	400	4.35	1.55	1–7	0.197 **	0.180 **	
	400	5.49	1.31	1–7	0.058	−0.058	−0.294

Note: Correlation coefficients shown in cells; ** *p* < 0.01; M = mean; SD = standard deviaton.

**Table 2:** Study Results from PROCESS Model 7 Analyses.

Effect	Estimate	*SE*	95% CI		*p*
			*LL*	*UL*	
Social Connection ^a^					
Intercept	1.766	0.645	0.498	3.033	0.007
Social Distancing	0.418	0.124	0.175	0.661	0.001
Iphone Use	0.299	0.112	0.079	0.519	0.008
Distancing Phone Use	−0.041	0.021	−0.083	0.000	0.053
Well-being ^b^ Intercept	6.330	0.294	5.753	6.908	<0.001
Social Distancing	0.093	0.051	−0.006	0.193	0.066
Social Connection	−0.294	0.041	−0.374	−0.214	<0.001

Note: Results based on the Preacher and Hayes (2008) PROCESS Model 7. SE = standard error; 95% CI = 95% confidence interval; LL = lower limit; UL = upper limit. ^a^ Social connection results (F_(3,374)_ = 9.47, *p* < 0.01 *R*^2^ = 0.07). ^b^ Well-being results (F_(2,375)_ = 26.06, *p* < 0.01 *R*^2^ = 0.12).

## Data Availability

The data presented in this study are available on request from the corresponding author. The data are not publicly available due to privacy issues included in the informed consent.

## References

[1] Center for Disease Control and Prevention (2020a). https://www.cdc.gov/coronavirus/2019-ncov/cases-updates/us-cases-deaths.html.

[2] Worldometer. www.worldometers.info/coronavirus/.

[3] Center for Disease Control and Prevention (2020b) Coronavirus Disease 2019 (Covid-19), Social Distancing: What is Social Distancing. https://www.cdc.gov/coronavirus/2019-ncov/prevent-getting-sick/social-distancing.html.

[4] Haleem A., Javaid M., Vaishya R. (2020). Effects of COVID-19 pandemic in daily life. Curr. Med. Res. Pr..

[5] McGinty E.E., Presskreischer R., Han H., Barry C.L. Psychological Distress and Loneliness Reported by US Adults in 2018 and April 2020. JAMA Research Letter, 3 June 2020. https://jamanetwork.com/journals/jama/fullarticle/2766941.

[6] Abel T., McQueen D. (2020). The Covid-19 pandemic calls for spatial distancing and social closeness: Not for social distancing!. Int. J. Public Health.

[7] Steinfield C., Ellison N.B., Lampe C. (2008). Social capital, self-esteem, and use of online social network sites: A longitudinal analysis. J. Appl. Dev. Psychol..

[8] Ahn D., Shin D. (2013). Is the social use of media for seeking connectedness or for avoiding social isolation? Mechanisms underlying media use and subjective well-being. Comput. Hum. Behav..

[9] DeWitte M. Instead of Social Distancing, Practice “Distant Socializing” Instead, Urges Stanford Psychologist. Stanford News. http://news.stanford.edu/2020/03/19/try-distant-socializing-instead/.

[10] Cudjoe T.K.M., Kotwal A.A. (2020). Social distancing amid a crisis in social isolation and Loneliness. JAGS.

[11] Chen C.-A., Chen D.-Y., Xu C. (2018). Applying Self-Determination Theory to Understand Public Employee’s Motivation for a Public Service Career: An East Asian Case (Taiwan). Public Perform. Manag. Rev..

[12] Deci E.L., Ryan R.M. (1985). Intrinsic Motivation and Self-Determination in Human Behavior.

[13] Demircioglu M.A. (2018). Examining the Effects of Social Media Use on Job Satisfaction in the Australian Public Service: Testing Self-Determination Theory. Public Perform. Manag. Rev..

[14] Evans D. (2020). How Zoom Became so Popular during Social Distancing. https://www.cnbc.com/2020/04/03/how-zoom-rose-to-the-top-during-the-coronavirus-pandemic.html.

[15] DiSalvo D. (2020). What the SARS Epidemic Can Teach Us About Relationships and Mental Health during the Coronavirus Pandemic. Forbes. https://www.forbes.com/sites/daviddisalvo/2020/03/27/what-the-sars-epidemic-can-teach-us-about-relationships-and-mental-health-during-the-coronavirus-pandemic/#68deb20aea4f.

[16] Roberts J., David M. (2016). My life has become a major distraction from my cell phone: Partner phubbing and relationship satisfaction among romantic partners. Comput. Hum. Behav..

[17] Schneider F.M., Hitzfeld S. (2019). I ought to put down that phone but I phub nevertheless: Examining the predictors of phubbing behavior. Soc. Sci. Comput. Rev..

[18] Conick H. (2020). How to Connect with Others in the Age of Social Distancing. Chicago News. https://news.uchicago.edu/story/how-connect-others-age-social-distancing.

[19] Van Orden K., Bower E., Lutz J., Silva C., Gallegos A.M., Podgorski C.A., Santos E.J., Conwell Y. (2020). Strategies to promote social connections among older adults during “social distancing” restrictions. Am. J. Geriatr. Psychiatry.

[20] Bian M., Leung L. (2015). Linking loneliness, shyness, smartphone addiction symptoms, and patterns of smartphone use to social capital. Soc. Sci. Comput. Rev..

[21] Primack B., Shensa A., Sidani J.E., Whaite E.O., Lin L.Y., Rosen D., Colditz J.B., Radovic A.M., Miller E. (2017). Social media use and perceived social isolation among young adults in the U.S. Am. J. Prev. Med..

[22] Bergman D., Behtell C., Gombojav N., Hassink S., Strange K.C. (2020). Physical distancing with social connectedness. Ann. Fam. Med..

[23] Twenge J.M. (2013). Does online social media lead to social connection or social disconnection. J. Coll. Character.

[24] Chan M. (2013). Mobile phones and the good life: Examining the relationships among mobile use, social capital and subjective well-being. New Med. Soc..

[25] Deters F.G., Mehl M.R. (2013). Does posting Facebook status updates increase or decrease loneliness? An online social networking experiment. Soc. Psychol. Pers. Sci..

[26] Roberts J.A., David M.E. (2019). The social media party: Fear of missing out (FoMO), social media intensity, connection, and well-being. Int. J. Hum. Comput. Interact..

[27] Cho J. (2015). Roles of smartphone app use in improving social capital and reducing social isolation. Cyberpsychol. Behav. Soc. Netw..

[28] Twenge J.M., Spitzburg B.H., Campbell W.K. (2019). Less in-person social interaction with peers among U.S. adolescents in the 21st century and links to loneliness. J. Soc. Pers. Relatsh..

[29] Verduyn P., Ybarra O., Reisbois M., Jonides J., Kross E. (2017). Do social networks sites enhance or undermine subjective well-being? A critical review. Soc. Issues Policy Rev..

[30] Wei R., Lo V. (2006). Staying Connected While on the Move. New Med. Soc..

[31] Lee R.M., Robbins S.B. (1995). Measuring belongingness: The social connectedness and the social assurance scales. J. Couns. Psychol..

[32] Kroenke K., Spitzer R.L., Williams J.B., Lowe B. (2009). An ultra-brief Screening Scale for Anxiety and depression: The PHQ-4. Psychosomatics.

[33] David M.E., Roberts J.A. (2017). Phubbed and alone: Phone snubbing, social exclusion, and attachment to social media. J. Assoc. Consum. Res..

[34] Preacher K.J., Hayes A.F. (2008). Asymptotic and resampling strategies for assessing and comparing indirect effects in multiple mediator models. Behav. Res. Methods.

[35] Hayes A.F. (2013). Introduction to Mediation, Moderation, and Conditional Process Analysis: A Regression-Based Approach.

[36] Hayes A.F. (2015). An index and test of linear moderated mediation. Multivar. Behav. Res..

[37] Wiederhold B.K. (2020). Social media use during social distancing. Cyberpsychol. Behav. Soc. Netw..

[38] Utz S., Muscanell N., Khalid C. (2015). Snapchat elicits more jealousy then Facebook: A comparison of Snapchat and Facebook use. Cyberpsychol. Behav..

[39] Galea S., Merchant R.M., Luriem N. (2020). The Mental Health Consequences of COVID-19 and Physical Distancing: The Need for Prevention and Early Intervention. JAMA Intern. Med..

